# Pressure ulcers and Charcot's definitions: report on two cases

**DOI:** 10.1590/S1516-31802008000400005

**Published:** 2008-07-03

**Authors:** Hélio Afonso Ghizoni Teive, Ricardo William Genaro Rodrigues de Campos, Renato Puppi Munhoz, Lineu César Werneck

**Keywords:** Pressure ulcer, Outcome and process assessment (Health Care), Persistent vegetative state, Amyotrophic lateral sclerosis, Úlcera de pressão, Avaliação de processos e resultados (Cuidados de Saúde), Estado vegetativo persistente, Esclerose amiotrófica lateral

## Abstract

**CONTEXT AND OBJECTIVE:**

Pressure ulcers are lesions caused by inadequate blood flow and tissue malnourishment secondary to prolonged pressure on skin, soft connective tissues, muscle and/or bones. The authors report two distinct clinical situations of severely compromised neurological patients who shared several predisposing factors for pressure ulcers, but with opposite outcomes regarding the development of pressure ulcers.

**CASE REPORTS:**

The first case was a young patient in a persistent vegetative state who developed pressure ulcers that resulted in secondary sepsis and death. The second case was a patient with a diagnosis of amyotrophic lateral sclerosis who, in spite of being bedridden for several months with severe immobility, never developed pressure ulcers. These intriguing contrary clinical situations had already been defined by Charcot in the nineteenth century, with his creation of the expression “*decubitus ominosus*”. He indicated that patients with amyotrophic lateral sclerosis usually did not develop this form of complication, as was illustrated by the cases presented here.

## RESUMO

**CONTEXTO E OBJETIVO:**

Escaras de decúbito (ED) são lesões decorrentes de fluxo sangüíneo inadequado, com desnutrição tecidual secundária à pressão prolongada da pele, tecido conjuntivo, músculos e/ou ossos. Os autores relatam dois casos de pacientes com grave comprometimento neurológico e com vários fatores predisponentes para o desenvolvimento de escaras de decúbito, porém com conseqüências opostas.

**RELATO DE CASOS:**

O primeiro caso, um jovem paciente em estado vegetativo persistente, desenvolveu extensas ED evoluindo para sepse e óbito. O segundo caso, o paciente com esclerose lateral amiotrófica (ELA), ficou longo período restrito ao leito com imobilidade acentuada, mas sem o aparecimento de ED. Estas duas situações clínicas já foram bem definidas por Charcot no século XIX, quando este criou o termo “*decubitus ominosus*” que se aplica ao primeiro caso, e quando definiu que pacientes com ELA raramente desenvolvem ED, fato observado no segundo caso.

## INTRODUCTION

Pressure ulcers, also known as decubitus ulcers, are lesions caused by inadequate blood flow and tissue malnourishment secondary to prolonged pressure on skin, soft connective tissues, muscle and/or bones.^1^ It is widely accepted that prolonged pressure leads to mechanical compression of capillary vessels, thereby resulting in tissue ischemia. In turn, this results in accumulation of toxic metabolites and cell death, which leads to ulceration and necrosis of adjacent skin and tissues.^1^

The region most frequently affected is the skin overlying the sacral region, in up to 67% of all cases. Nevertheless, pressure ulcers can occur in other areas such as in the lower limbs, elbows and occipital region.^1,2^ These areas are susceptible to external factors (compression and friction), but other direct and indirect factors may also play a role. Some examples are the clinical conditions that lead to malnutrition, anemia, fever, peripheral vascular disease and diabetes mellitus and the abnormal postures resulting from spasticity or dystonia.^1,2^ In addition, pressure ulcers may also be associated with particular risk factors such as old age (> 70 years of age), urinary or fecal incontinence and consciousness disorders.^1-4^

## OBJECTIVE

The aim of this paper was to report on two distinct clinical situations of severely compromised neurological patients who shared several predisposing factors for pressure ulcers, but with completely different outcomes.

## CASE REPORTS

**Case 1.** This was a 19-year-old male patient who was admitted to the emergency room with clinical signs of intracranial hypertension. Clinical assessment showed that the condition was secondary to cerebral aqueduct stenosis due to acute bacterial meningitis. The patient had already undergone neurosurgical procedures, including ventriculoperitoneal shunting, several revisions of this procedure, external ventricular shunting and antibiotic treatment for coagulase-negative Staphylococcus aureus infection. The patient did not recover consciousness and was diagnosed as being in a persistent vegetative state with spastic quadriparesis. After two weeks, he developed extensive pressure ulcers in the sacral and right trochanteric areas, with severe secondary bacterial infection and deep tissue erosion that left the right hip joint exposed. The symptoms worsened, and he developed sepsis and died 90 days after admission.

**Case 2.** This was a 58-year-old male patient who was admitted with a four-year history of amyotrophic lateral sclerosis (ALS). Following an episode of bronchial infection, he developed respiratory distress. This led to orotracheal intubation and mechanical ventilation, initially in the intensive care unit, then in the ward and eventually at home. At his last follow-up he was quadriplegic, with severe generalized muscle atrophy but still able to maintain some degree of communication with his relatives through eye contact and eye movements. Although bedridden for a prolonged period of time, he had never developed pressure ulcers.

## DISCUSSION

Both cases presented here are frequent clinical situations among neurological patients who remain bedridden for a long period of time.^2^ Redelings et al.^5^ studied the mortality rates associated with pressure ulcers and concluded that these complications were frequently associated with fatal sepsis, commonly occurring in patients with neurodegenerative processes and other disabling chronic disorders. This correlation had already been established in the nineteenth century by Professor Jean-Martin Charcot. Over the course of his studies on pressure ulcers, he established what he termed the “neurotrophic theory”, in which a lesion in the central nervous system was thought to be the key factor in the development of pressure ulcers.^6^ Charcot also created the expression “*decubitus ominosus*”, to describe patients who suffered from severe neurological diseases and developed pressure ulcers particularly over the sacral region, thereby indicating short survival.^7^ In addition, he also suggested that pressure ulcers could lead to the occurrence of systemic infections, with “gangrenous metastases” to the lungs and could have predictive value regarding a fatal outcome.^7^ This ominous outcome could be observed with regard to the first patient described here.

One intriguing finding is that pressure ulcers are either absent or only rarely occur in patients with ALS, even after such patients have spent long periods confined to bed and presenting severe motor limitations, as seen in the second patient described here. Again, Charcot described this paradox more than a century ago^8^ and hypothesized several explanations for such a phenomenon. The most commonly accepted explanation is that both superficial and deep sensitivity are preserved in patients with ALS. However, other hypotheses have been presented, mostly relating to specific skin changes in ALS patients such as the “late-return phenomenon” (when the skin of such patients is stretched, it tends to return to its normal position rather slowly).^9-11^ Kolde et al.,^12^ among other authors, further demonstrated disorganization of the elastin and collagen layers of the skin of ALS patients, with beta-amyloid deposits. Ono et al.^13^ reported that the expression of laminin 1 was elevated in the skin of patients with ALS, a finding that might also contribute towards the change in skin mechanical properties in these patients.

## CONCLUSION

In view of these and other observations, we can safely conclude that the definitions established by Charcot in the nineteenth century are still extremely valuable.^7,8^

## References

[1] Bansal C, Scott R, Stewart D, Cockerell CJ (2005). Decubitus ulcers: a review of the literature. Int J Dermatol.

[2] Stausberg J, Kröger K, Maier I, Niebel W, Schneider H (2005). Häufigkeit des Dekubitus in einem Universitätsklinikum. Kombination von Routinedokumentation und Querschnittstudie. [Frequency of decubitus ulcer in patients of a university medical center. Combination of routine documentation and cross-sectional study].. Dtsch Med Wochenschr.

[3] Karlsson AK (2006). Autonomic dysfunction in spinal cord injury: clinical presentation of symptoms and signs. Prog Brain Res.

[4] Post MW, Dallmeijer AJ, Angenot EL, van Asbeck FW, van der Woude LH (2005). Duration and functional outcome of spinal cord injury rehabilitation in the Netherlands. J Rehabil Res Dev.

[5] Redelings MD, Lee NE, Sorvillo F (2005). Pressure ulcers: more lethal than we thought?. Adv Skin Wound Care.

[6] Levine JM (1992). Historical perspective: the neurotrophic theory of skin ulceration. J Am Geriatr Soc.

[7] Levine JM (2005). Historical perspective on pressure ulcers: the decubitus ominosus of Jean-Martin Charcot. J Am Geriatr Soc.

[8] Charcot JM, Charctor JM (1962). Lecture XIII. On amyotrophic lateral sclerosis. Symptomatology. Lectures on the diseases of the nervous system.

[9] Parish LC, Smith G, Collins E (1978). Decubitus ulcers and amyotrophic lateral sclerosis. Lancet.

[10] Ono S (2000). The skin in amyotrophic lateral sclerosis. Amyotroph Lateral Scler Other Motor Neuron Disord.

[11] Ono S, Toyokura Y, Mannen T, Ishibashi Y (1988). “Delayed return phenomenon” in amyotrophic lateral sclerosis. Acta Neurol Scand.

[12] Kolde G, Bachus R, Ludolph AC (1996). Skin involvement in amyotrophic lateral sclerosis. Lancet.

[13] Ono S, Imai T, Shimizu N, Nagao K (2000). Increased expression of laminin 1 in the skin of amyotrophic lateral sclerosis. Eur Neurol.

